# Comparison of Open and Closed Reduction for Developmental Dysplasia of the Hip: A Retrospective Evaluation of Acetabular Development and Complications

**DOI:** 10.3390/jcm15197458

**Published:** 2026-09-25

**Authors:** Kutbettin Dinçer, Mehmet Akif Şahin

**Affiliations:** Department of Orthopedics and Traumatology, Gazi Yaşargil Training and Research Hospital, University of Health Sciences, Diyarbakır 21070, Türkiye

**Keywords:** developmental dysplasia of the hip, avascular necrosis, acetabular index, open reduction, closed reduction, osteotomy, Tönnis classification

## Abstract

**Background/Objectives:** Developmental dysplasia of the hip (DDH) is a common pediatric musculoskeletal disorder, and the optimal treatment approach remains controversial. This study compared open and closed reduction in terms of acetabular development, avascular necrosis (AVN) of the femoral head, and the need for subsequent pelvic osteotomy. **Methods:** This retrospective cohort study evaluated 75 patients (95 hips; 48 right, 47 left) treated for DDH at a tertiary referral center. We performed open reduction in 63 hips and closed reduction in 32. We performed arthrography in 50 hips (52.6%) with a positive Ortolani test to assess reduction quality. We assessed acetabular remodeling using the acetabular index (AI) at 6, 12, and 36 months postoperatively. Dislocation severity was graded using the Tönnis classification, and AVN was assessed using the Kalamchi–MacEwen criteria. Statistical analyses included chi-square tests, independent-samples *t*-tests, one-way ANOVAs, and Pearson correlation tests, with significance set at *p* < 0.05. **Results:** The mean age at surgery was 12.83 ± 5.13 months (range: 6–24 months), and the mean follow-up was 37.78 ± 8.07 months (range: 36–60 months). AVN was observed in 7/63 hips (11.1%) after open reduction and in 0/32 hips after closed reduction (two-sided Fisher exact test, *p* = 0.091). Among the seven AVN-affected hips, four were Kalamchi–MacEwen grade I, two were grade II, and one was grade III; no grade IV case was observed. Secondary pelvic osteotomy was performed in 13/63 hips (20.6%) after open reduction and 2/32 hips (6.3%) after closed reduction. AI improved during follow-up, decreasing from 38.31 ± 4.47° at 6 months to 31.59 ± 4.28° at 12 months and 23.65 ± 6.03° at 36 months. Higher Tönnis grades were associated with higher 36-month AI values (*p* = 0.018). Older age at surgery correlated positively with AI at 12 months (*p* = 0.038) and 36 months (*p* < 0.001). **Conclusions:** Open reduction was associated with higher rates of AVN and secondary pelvic osteotomy; however, treatment selection was based on reducibility and arthrographic findings, precluding causal interpretation. Older age at treatment and greater dislocation severity were associated with less favorable acetabular remodeling. Arthrography-assisted closed reduction remains a reasonable option when a stable concentric reduction is achievable, although neither method demonstrated clear superiority.

## 1. Introduction

Developmental dysplasia of the hip (DDH) is a developmental disorder that ranges from simple acetabular dysplasia to complete dislocation of the femoral head. This condition affects 1–1.5% of newborns [1]. If not treated early, it may lead to gait disturbance, limb-length discrepancy, and early-stage coxarthrosis, eventually necessitating total hip arthroplasty [2,3]. Treatment aims to achieve complete reduction of the femoral head into the underdeveloped acetabulum and to maintain that reduction. This approach promotes the development of both the femoral head and acetabulum [4]. The main factors determining treatment are the age at diagnosis and the severity of dislocation. In infants younger than six months, bracing is the preferred method for treating hip dislocation. Treatment with the Pavlik harness is highly effective in the short and long term [5]. The success rate of Pavlik harness treatment is around 80% [6]. For patients older than six months who do not benefit from the Pavlik harness or other conservative methods, closed reduction (CR) under anesthesia followed by spica casting is the standard treatment [7,8]. Causes of failed closed reduction may include soft-tissue factors such as an inverted labrum, ligamentum teres hypertrophy, or a joint capsule constricting the acetabulum. In such cases, open reduction becomes necessary [9].

One of the most serious complications of both surgical techniques is avascular necrosis (AVN) of the femoral head [10]. This condition can lead to femoral head deformity, limb shortening, and early hip degeneration. The reported incidence of AVN varies widely, from 6% to 48%, depending on classification systems, follow-up duration, and patient populations [11]. In the meta-analysis by Novais et al. [12], the incidence of AVN identified during follow-up was higher after open reduction (19%) than after closed reduction (8%). However, the literature remains conflicting on this issue. In addition, insufficient acetabular remodeling may necessitate acetabular reconstruction, such as femoral or pelvic osteotomy [13].

The acetabular index (AI) is a valuable parameter for assessing the acetabular osseous roof and for monitoring acetabular development after concentric reduction [14]. Factors that influence acetabular remodeling include age at reduction, dislocation severity at diagnosis (Tönnis type), reduction method, and femoral head avascular necrosis [15]. However, the precise effects of these factors on acetabular development remain unclear, and the timing and indications for subsequent osteotomy are debated [16].

This single-center retrospective study compared open and closed reduction for DDH with respect to AVN occurrence, AI-based acetabular remodeling, and the need for secondary pelvic osteotomy. Given the retrospective design, treatment-selection process, and low number of AVN events, we described clinical associations rather than identifying independent predictors or inferring causal treatment superiority.

## 2. Materials and Methods

### 2.1. Study Design and Ethical Approval

The Ethics Committee of Gazi Yaşargil Training and Research Hospital approved this retrospective study (protocol code 175, approval date 24 April 2026). We conducted the study in accordance with the Declaration of Helsinki. We reviewed medical records and radiographs of children aged 6–24 months who underwent open or closed reduction at our hospital from 2020 to 2024.

### 2.2. Study Population

We included 75 patients (95 hips) who met our criteria. Inclusion criteria were radiographically confirmed DDH, definitive treatment with open or closed reduction, complete clinical and radiographic data at 6, 12, and 36 months postoperatively, and a minimum total follow-up of 36 months from surgery. All included hips underwent primary reduction at our institution. Patients who underwent concomitant femoral or pelvic osteotomy at the index procedure were excluded. We excluded 20 patients: 5 with teratologic or syndromic dislocation, 5 initially treated or followed at outside centers, 6 with incomplete clinical or radiographic follow-up, and 4 with a concomitant osteotomy during the index procedure. We used the Tönnis classification to grade dislocation severity at the time of diagnosis [17]. We selected the Tönnis classification because it was consistently available on preoperative radiographs in this retrospective cohort, allowing uniform classification across all included hips; however, future studies may consider the IHDI (International Hip Dysplasia Institute) classification for comparison and reproducibility.

### 2.3. Surgical Technique

All procedures were performed by the same surgeon under general anesthesia. We assessed hip reducibility with the Ortolani test under anesthesia. We performed arthrography in 50 hips (52.6%) with a positive Ortolani test to assess reduction quality and determine whether any structures prevented reduction. We did not perform arthrography in hips with a negative Ortolani test; we directed these hips to open reduction based on clinical assessment. The arthrographic procedure was performed under fluoroscopic guidance with the patient supine, the hip abducted to 45°, and an 18-gauge needle inserted just below the adductor tendon, 2 cm inferior to the pubic symphysis, directed toward the ipsilateral shoulder. Saline was injected under fluoroscopic guidance to confirm intra-articular needle placement. Subsequently, a radiopaque contrast agent (diatrizoic acid/Urografin) was injected into the hip joint for assessment. Arthrography was used to evaluate the position of the femoral head within the acetabulum, the adequacy of reduction, and the presence of an inverted limbus, pulvinar tissue, and hypertrophy of the ligamentum teres.

After arthrography, medial pooling of less than 5 mm indicated a stable concentric reduction (Figure 1). We selected closed reduction for patients with a positive Ortolani test and medial pooling of less than 5 mm on arthrography.

We defined the safe zone using Ramsey’s criteria [18]. Adductor tenotomy was performed when the hip dislocated within the 30–50° abduction range (safe-zone criterion not met). It was not performed in hips that were concentrically reduced within the safe zone. For closed reduction, we performed the reduction under fluoroscopic guidance and applied a pelvic-pedal cast at 90° flexion, 40° abduction, and 15° internal rotation (Figure 2).

Open reduction was performed on hips with a negative Ortolani test or medial pooling exceeding 5 mm on arthrography, as well as on hips in which a stable, concentric closed reduction could not be maintained intraoperatively (Figure 3). We performed the procedure via an anterior Smith-Petersen approach. During open reduction, the ligamentum teres was excised, pulvinar tissue was removed, the transverse acetabular ligament was released, and iliopsoas and adductor tenotomies were performed as indicated by intraoperative assessment. The inverted labrum, if present, was reduced. Capsulorrhaphy was then performed. To maintain postoperative reduction, we applied a pelvic-pedal cast with the hip in 30° flexion, 30° abduction, and 15° internal rotation (Figure 4 and Figure 5). The lower flexion angle in the open-reduction cast (30° vs. 90°) reflects the increased capsular and soft-tissue stability achieved after open surgical repair; a more flexed position is not required to maintain reduction and has been associated with elevated AVN risk after open reduction.

### 2.4. Postoperative Follow-Up

After surgery, patients were recalled at 6 weeks to assess the need for cast revision. In some patients, casts were changed at 6 weeks because of breakdown or weight gain. At the end of the third month, casts were discontinued after AP pelvis radiographs were obtained in all patients. To maintain reduction, patients wore a Denis Browne orthosis for 6 weeks, with the hips in 30° abduction and 15° internal rotation. We observed no redislocation in any hip during follow-up after closed or open reduction.

We measured AI on standard AP pelvis radiographs at 6, 12, and 36 months postoperatively in all patients (Figure 6). We did not measure the acetabular index at 60 months; the 36-month measurement was the final planned radiographic assessment for this outcome. All included hips had AI measurements at each of the three prespecified time points (6, 12, and 36 months), and total follow-up ranged from 36 to 60 months. We obtained all 36-month AI measurements before any secondary pelvic osteotomy. Secondary pelvic osteotomy was considered and performed only after completion of the 36-month AI assessment, when residual acetabular dysplasia was identified on clinical and serial radiographic evaluation (Figure 7a,b). On the final available AP pelvis radiographs, femoral-head AVN was evaluated using the Kalamchi–MacEwen classification [19]. Two observers experienced in pediatric hip radiography assessed AVN independently and resolved disagreements by consensus. We did not calculate formal interobserver agreement for categorical grading (kappa) or intra- and interobserver reliability for AI measurements (intraclass correlation coefficients) in this retrospective study; therefore, we report no reliability coefficients. During follow-up, we considered secondary acetabuloplasty and pelvic osteotomy when clinical and serial radiographic assessment indicated insufficient acetabular remodeling. We individualized the indication and timing of this decision and did not prospectively protocolize it.

### 2.5. Statistical Analysis

We analyzed data from 95 hips in 75 patients using IBM SPSS Statistics version 27.0. Because we included both hips from some patients, we used the hip as the primary unit of analysis and did not adjust for patient-level clustering. After reviewing the descriptive findings, we conducted normality tests to determine appropriate methods for comparing continuous variables. We used the chi-square test for categorical variables when expected cell counts were adequate; Fisher’s exact test was used for sparse 2 × 2 comparisons. We used the independent-samples t test for comparisons between dichotomous variables and normally distributed continuous variables, one-way ANOVA for comparisons among three-category variables and normally distributed continuous variables, and Pearson correlation analysis to evaluate relationships between normally distributed variables. Statistical significance was set at *p* < 0.05 for all analyses.

## 3. Results

The study included 75 patients and 95 hips (48 right, 47 left). The mean age at surgery was 12.83 ± 5.13 months (range: 6–24 months), and the mean follow-up duration was 37.78 ± 8.07 months (range: 36–60 months). Open reduction was performed in 63 hips (66.3%), and closed reduction in 32 hips (33.7%). Arthrography was performed in 50 hips (52.6%). According to the Tönnis classification, 7 hips (7.4%) were type 2, 39 hips (41.1%) were type 3, and 49 hips (51.6%) were type 4. No hips were Tönnis type 1.

Femoral-head AVN was observed in seven of 95 hips (7.4%), whereas no radiographic evidence of AVN was identified during the available follow-up in 88 hips (92.6%). All seven AVN-affected hips underwent open reduction (7/63, 11.1%), and no AVN events were observed after closed reduction (0/32, 0%). A two-sided Fisher exact test yielded *p* = 0.091; therefore, this unadjusted difference was not statistically significant at the prespecified threshold of *p* < 0.05. According to the Kalamchi–MacEwen classification, four AVN-affected hips were grade I (57.1%), two were grade II (28.6%), and one was grade III (14.3%); no grade IV AVN was observed. Given the algorithm-based treatment selection and the small number of events, these findings are descriptive associations rather than causal estimates (Table 1).

During follow-up, we performed secondary pelvic osteotomy in 15 hips (15.8%): 13/63 hips (20.6%) in the open-reduction group and 2/32 hips (6.3%) in the closed-reduction group. The between-group comparison was exploratory (Fisher exact test, *p* = 0.081). We individualized the indication and timing of osteotomy during follow-up based on clinical and serial radiographic assessment of acetabular remodeling, and we did not standardize them prospectively. Accordingly, osteotomy should be interpreted as a surgeon-dependent secondary clinical outcome rather than an independent comparative endpoint (Table 2). We obtained all 36-month AI measurements before secondary pelvic osteotomy; therefore, the reported 36-month AI results were not affected by the subsequent osteotomy.

Mean AI values at 6, 12, and 36 months were 38.31 ± 4.47°, 31.59 ± 4.28°, and 23.65 ± 6.03°, respectively, indicating marked acetabular remodeling during follow-up. At 6 months, mean AI values were 34.00 ± 4.16° in Tönnis type 2, 39.31 ± 4.58° in type 3, and 38.12 ± 4.10° in type 4 hips. AI values in Tönnis type 2 patients were significantly lower; 6-month AI values differed significantly among groups (*p* = 0.013). At 36 months, AI values were significantly higher in Tönnis type 4 hips (25.18 ± 6.38°) than in type 2 (19.57 ± 3.41°) and type 3 hips (22.46 ± 5.39°) (*p* = 0.018). This finding indicates less favorable residual acetabular development in hips with more severe initial dislocation (Table 3).

Age at surgery was positively correlated with AI at 12 months (*p* = 0.038) and 36 months (*p* < 0.001), suggesting that delayed surgical intervention is associated with less favorable acetabular remodeling. At 12 months, AI was significantly higher in hips that later required osteotomy (36.07 ± 4.23°) than in those that did not (30.75 ± 3.76°) (*p* < 0.001), whereas no significant difference was observed at 6 months (*p* = 0.401). The 12-month acetabular index may serve as an early marker of persistent dysplasia; however, interpret this finding with caution, as the indication for osteotomy was not standardized (Table 4).

## 4. Discussion

As DDH treatment techniques have evolved, the choice between open and closed reduction remains a critical clinical decision. In this retrospective cohort, AVN and secondary pelvic osteotomy rates were higher in hips treated with open reduction. However, these findings must be interpreted in the context of substantial treatment selection bias, because allocation to open versus closed reduction was based on reducibility, arthrographic findings, and baseline severity rather than randomization. Accordingly, our data describe associations within a selected cohort and do not establish that open reduction itself caused poorer outcomes.

In our study, the AVN rate was 7.4% (7/95 hips), whereas rates reported in the literature range from 0% to 67%. Our observed rate was lower than several reported rates; however, interpret this comparison cautiously because AVN definitions, follow-up durations, and patient populations differed [11,20,21]. All 7 AVN cases occurred in patients who underwent open reduction (7/63, 11.1%). No AVN was observed after closed reduction (0/32). The two-sided Fisher exact test yielded *p* = 0.091; this difference was not statistically significant. Because we identified only seven AVN cases, statistical power was insufficient to support an adjusted multivariable analysis. We could not reliably identify independent risk factors, and no variable should be designated as an independent predictor.

In the meta-analysis by Novais et al., AVN was reported in 19% of open reductions and 8% of closed reductions [12]. Pospischill et al. [10] reported AVN in 40% of cases, particularly when osteotomy and open reduction were performed in the same session. Our findings are directionally consistent with that literature but should be viewed as hypothesis-generating and broadly confirmatory rather than definitive or practice-changing.

The literature remains controversial regarding AVN risk factors. The study dataset did not include the presence or absence of the femoral-head ossific nucleus, so we did not analyze it as a cohort-specific risk factor. Although previous studies have assessed a possible association between ossific-nucleus status and AVN [22,23,24], this cohort cannot provide evidence on this issue. The absence of this variable is a limitation of the available retrospective data.

The progressive decrease in AI over 36 months (from 38.31° at 6 months to 23.65° at 36 months) aligns with the well-known pattern of acetabular remodeling after complete reduction [13,25]. The significant association between higher Tönnis grade and higher AI values at 36 months underscores that dislocation severity at diagnosis is an important determinant of residual acetabular development. The higher 36-month AI values in Tönnis type 4 hips suggest poorer residual acetabular development and more limited remodeling potential in the most severely dislocated hips, rather than a slower biological remodeling rate per se. Importantly, we obtained all 36-month AI measurements before secondary pelvic osteotomy. Thus, the observed reduction in AI over the study period was not attributable to the radiographic effect of osteotomy.

The positive correlation between age at surgery and AI at 12 months (*p* = 0.038) and 36 months (*p* < 0.001) suggests that delayed reduction may be linked to less favorable acetabular remodeling. This observation aligns with Albinana et al. [25], who identified AI at 1 year after surgery as a strong predictor of residual dysplasia, and with Shin et al. [16], who reported that hips with AI ≥32° and a center-edge angle ≤14° at 3 years of age benefited significantly from osteotomy.

In our study, the secondary pelvic osteotomy rate was 15.8% (15/95 hips), which falls within previously reported ranges of 11–35% [7,26]. Within-group rates were 20.6% (13/63) after open reduction and 6.3% (2/32) after closed reduction. The finding that 12-month AI was significantly higher in hips that later underwent pelvic osteotomy (36.07° vs. 30.75°, *p* < 0.001), whereas 6-month AI did not differ (*p* = 0.401), may be clinically informative. Because the indication for osteotomy was not prospectively standardized, this association should be regarded as exploratory rather than a validated prediction rule.

Our study has several limitations. First, it is retrospective and subject to selection bias. Second, clinicians chose between open and closed reduction based on clinical and intraoperative findings rather than random assignment. Third, the follow-up duration (mean 37.78 months, range 36–60 months) may not capture late-developing AVN or residual dysplasia; the results should therefore be considered mid-term radiographic outcomes. We measured the acetabular index at 6, 12, and 36 months only; we did not perform a 60-month radiographic assessment. Fourth, we did not perform formal intra- and interobserver reliability analyses; we did not calculate intraclass correlation coefficients for AI measurement or kappa statistics for Tönnis and Kalamchi–MacEwen grading in this retrospective study. Fifth, the low number of AVN events (n = 7) precluded multivariable adjustment and limited statistical power. Sixth, although all 36-month AI measurements preceded secondary pelvic osteotomy, the indication and interval between the 36-month assessment and osteotomy were not prospectively standardized. Accordingly, osteotomy remains a surgeon-dependent exploratory secondary clinical outcome. Seventh, we included both hips from some patients and did not adjust for within-patient clustering; consequently, hip-level comparisons may be overconfident. Eighth, the dataset did not include ossific-nucleus status or quantitative counts of adjunctive tenotomies.

An additional limitation is that the present study does not evaluate other treatment strategies, such as traction, gradual reduction, or closed reduction after routine adductor tenotomy, as distinct comparative pathways. Therefore, the current data do not support a broad claim that arthrography-assisted closed reduction should be the universal initial treatment for all children with DDH. Rather, our findings support the narrower conclusion that when a hip is demonstrably reducible and a concentric reduction can be achieved safely, a closed approach may be associated with favorable mid-term radiographic outcomes in a selected subgroup.

Despite these limitations, the study provides clinically relevant descriptive data on the occurrence of AVN, acetabular remodeling, and secondary surgery following reduction for DDH.

## 5. Conclusions

This retrospective study of 75 patients and 95 hips treated for DDH found that AVN and secondary pelvic osteotomy occurred more often in hips managed with open reduction; however, these associations were unadjusted and observed in a selected cohort. Because treatment allocation depended on reducibility and arthrographic findings, these differences should not be interpreted as causal treatment effects. Older age at surgery and higher Tönnis grade were associated with less favorable acetabular remodeling. The 12-month acetabular index may be a useful early marker of persistent dysplasia, although this observation requires confirmation in studies with standardized indications for secondary surgery. When stable concentric reduction can be achieved closed, arthrography-assisted closed reduction remains a reasonable option; nevertheless, further prospective, multicenter studies with longer follow-up are needed before firm comparative recommendations can be made.

## Figures and Tables

**Figure 1:**
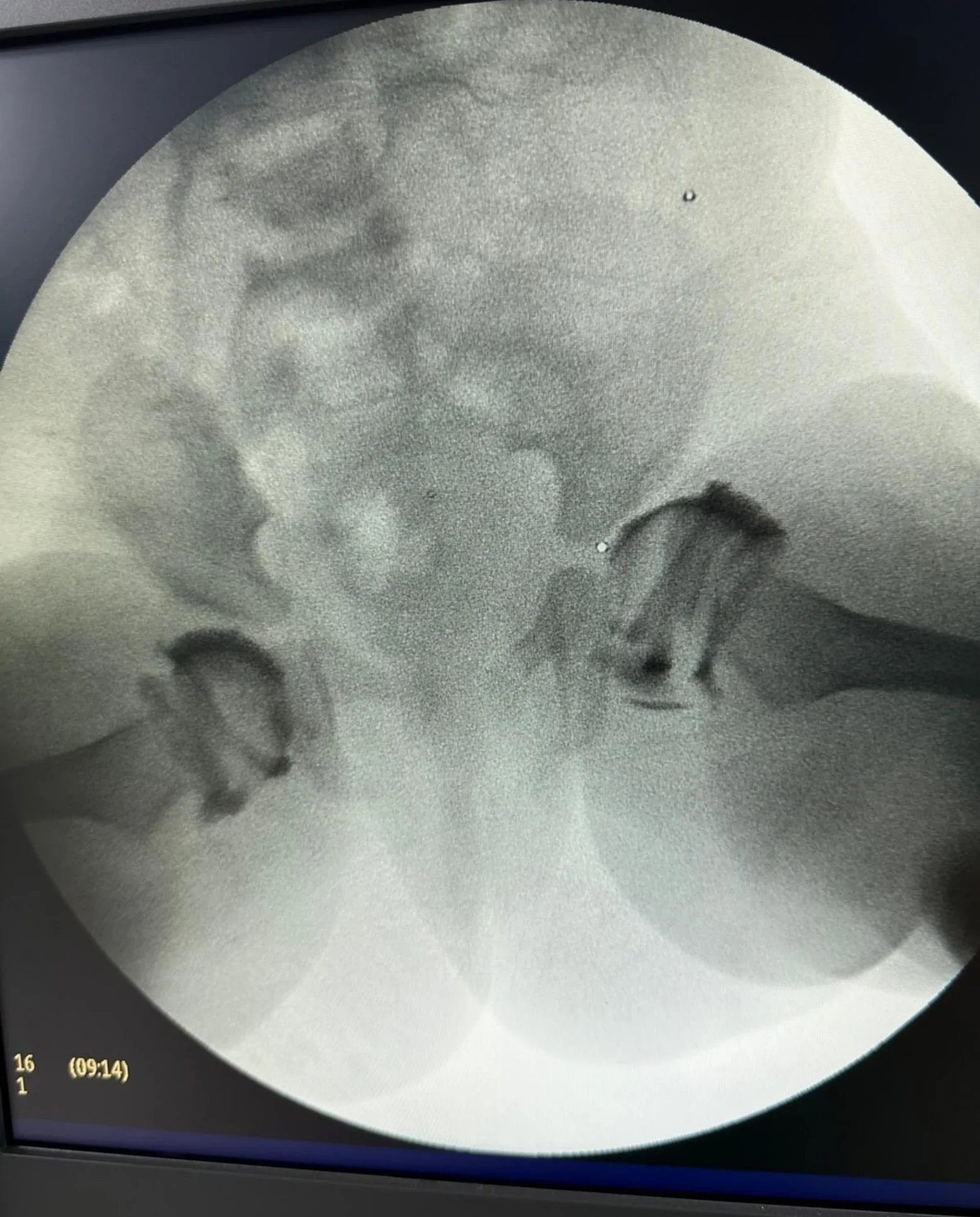
Arthrography image from the surgery of a 12-month-old child with developmental hip dysplasia. Closed reduction was decided upon because the medial pooling on bilateral hip arthrography was less than 5 mm.

**Figure 2:**
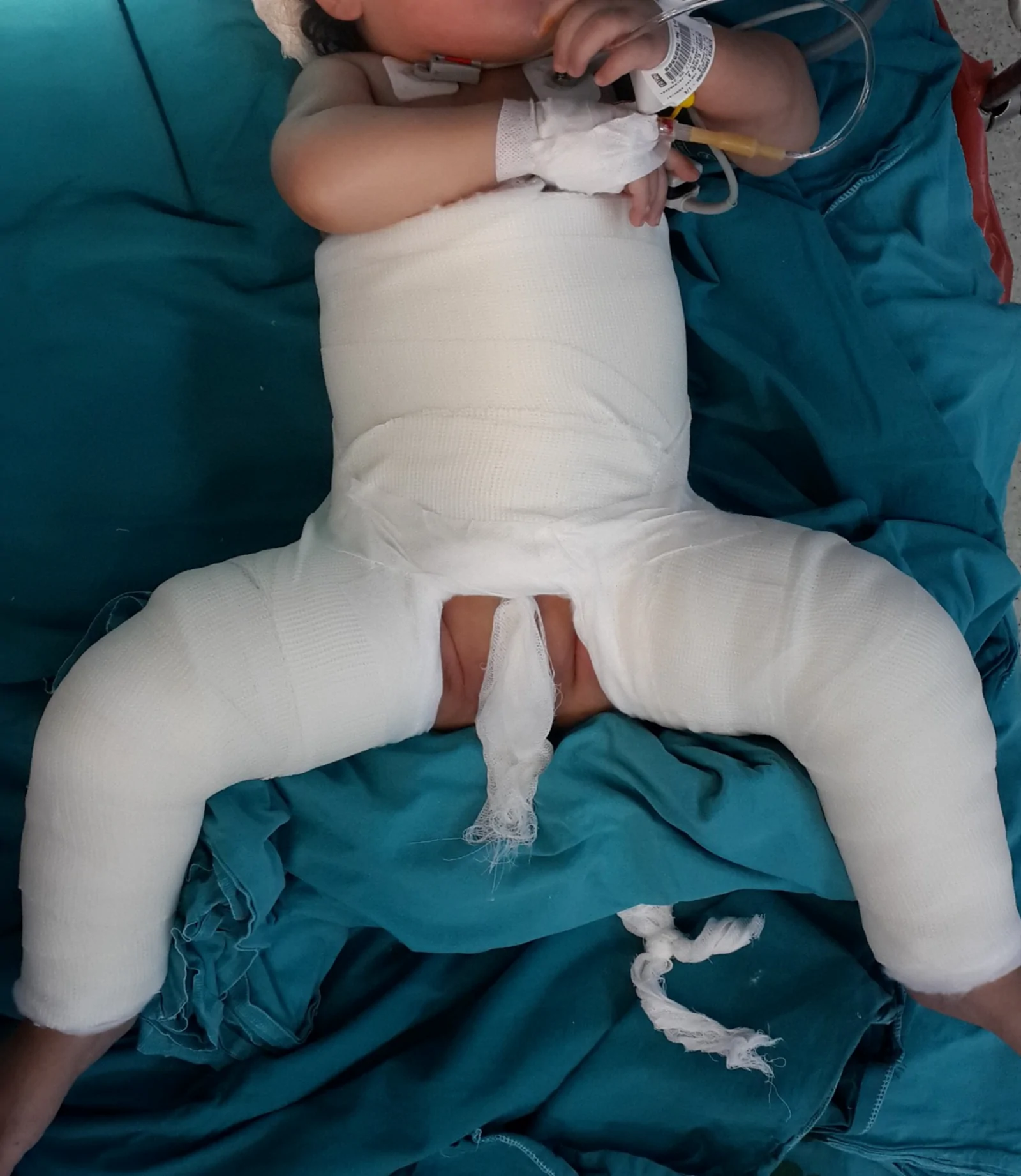
Image of a 12-month-old child with bilateral developmental hip dysplasia after closed reduction and hip spica casting.

**Figure 3:**
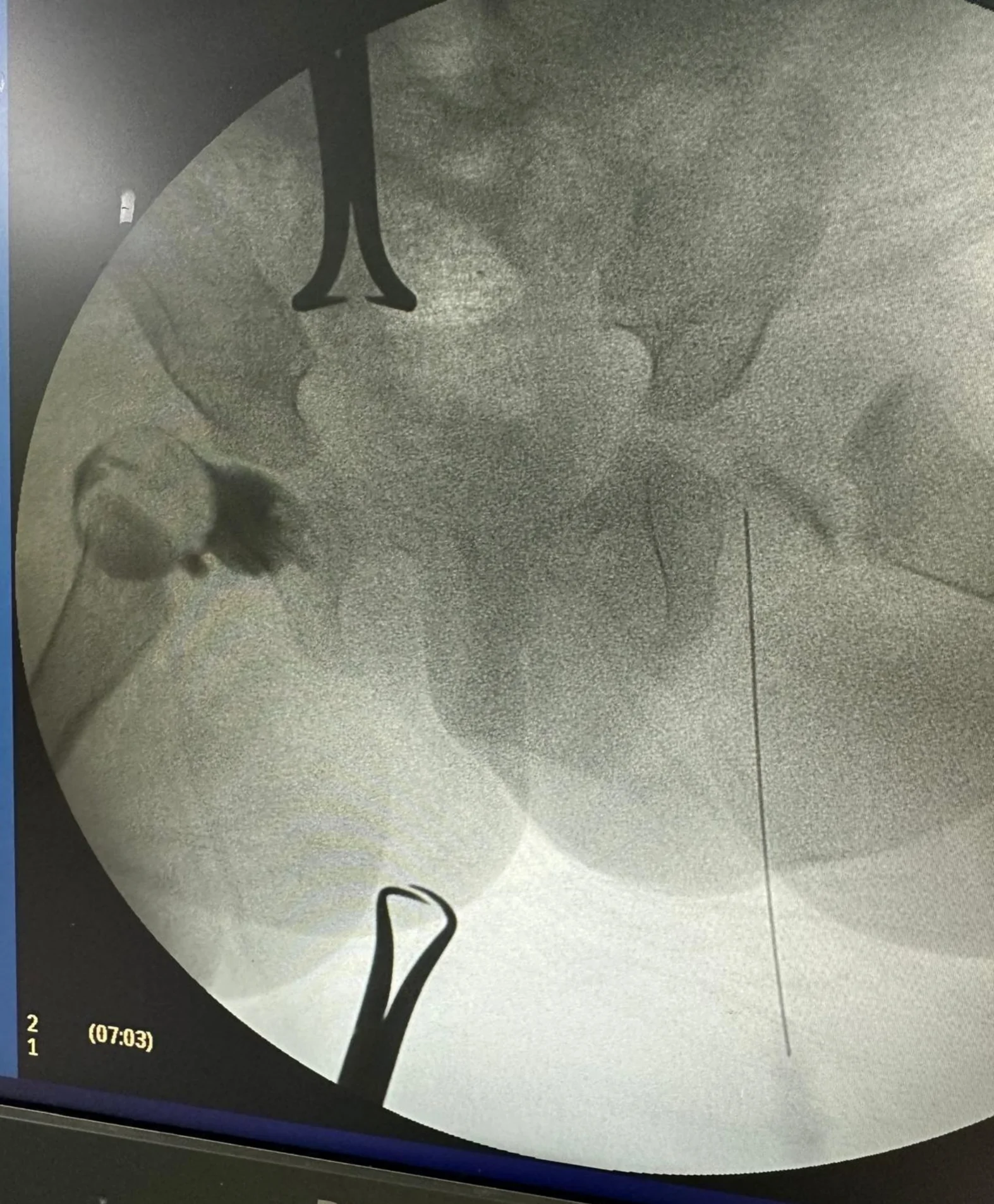
Arthrography image from the surgery of a 15-month-old child with developmental hip dysplasia in the right hip. The decision for open reduction was made because the medial pooling on the right hip arthrography was greater than 5 mm.

**Figure 4:**
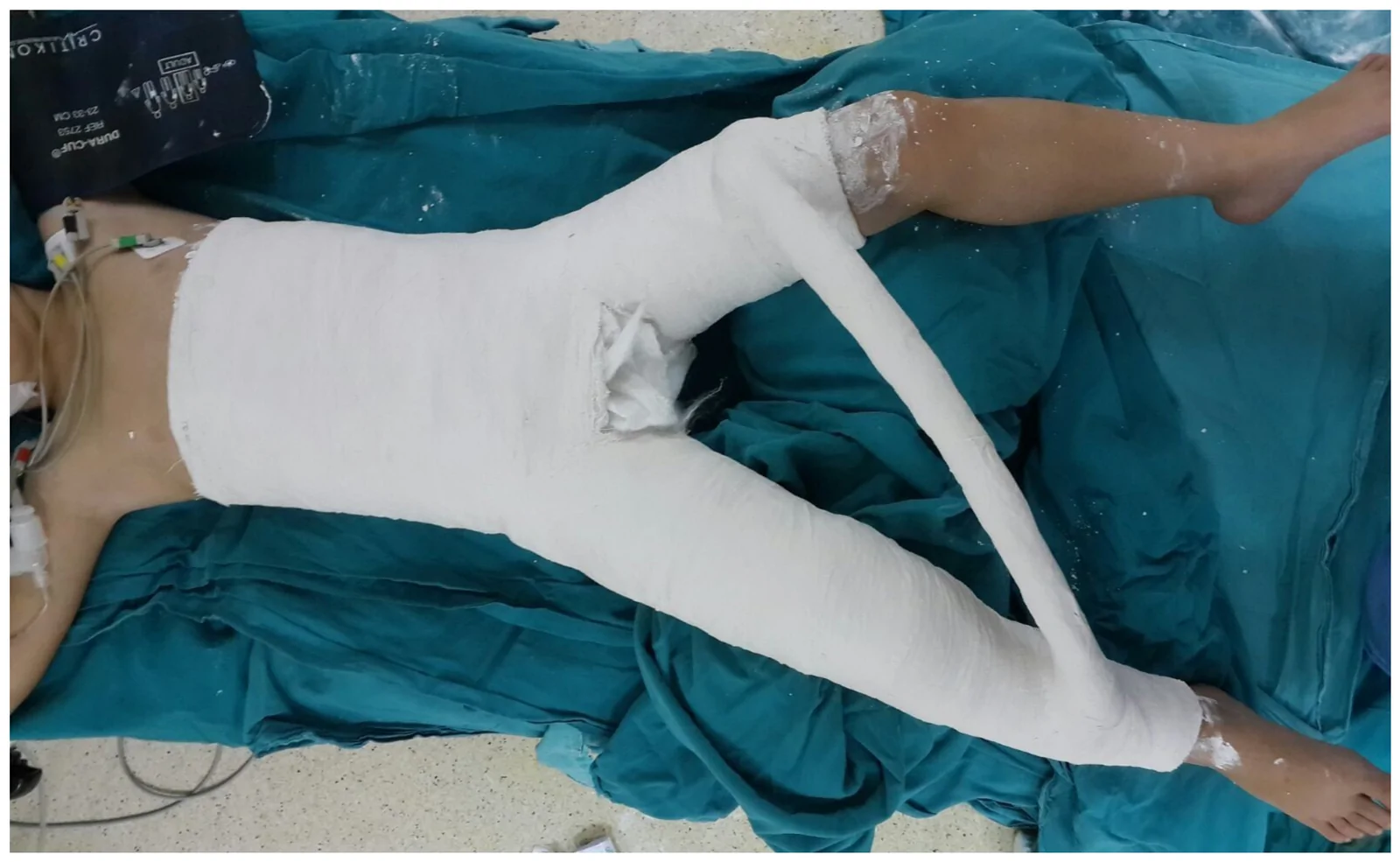
Image of an 18-month-old child with right hip Tönnis grade 4 developmental hip dysplasia after open reduction and hip spica casting.

**Figure 5:**
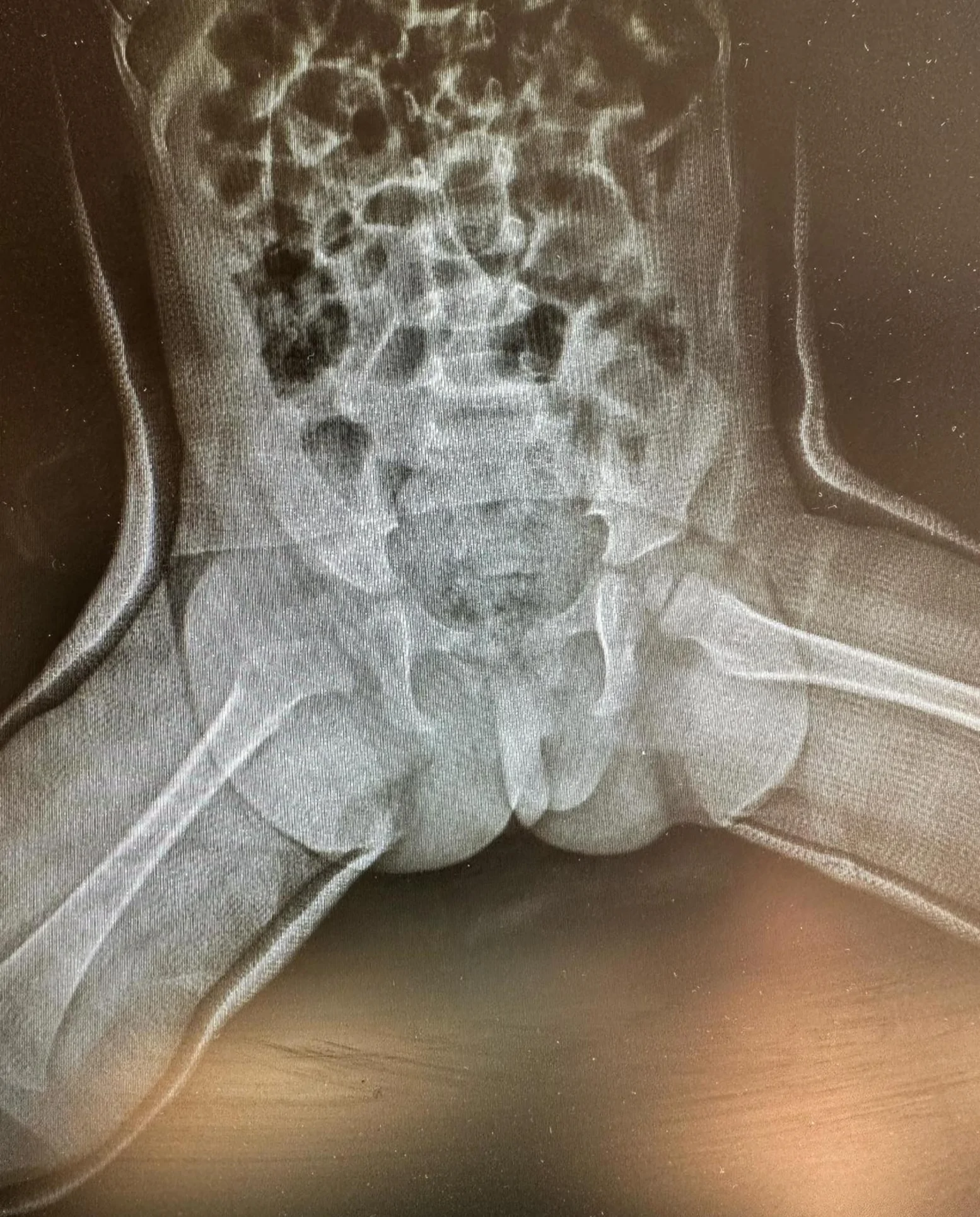
Radiographic image of an 18-month-old child with right hip Tönnis grade 4 developmental hip dysplasia following open reduction and hip spica casting.

**Figure 6:**
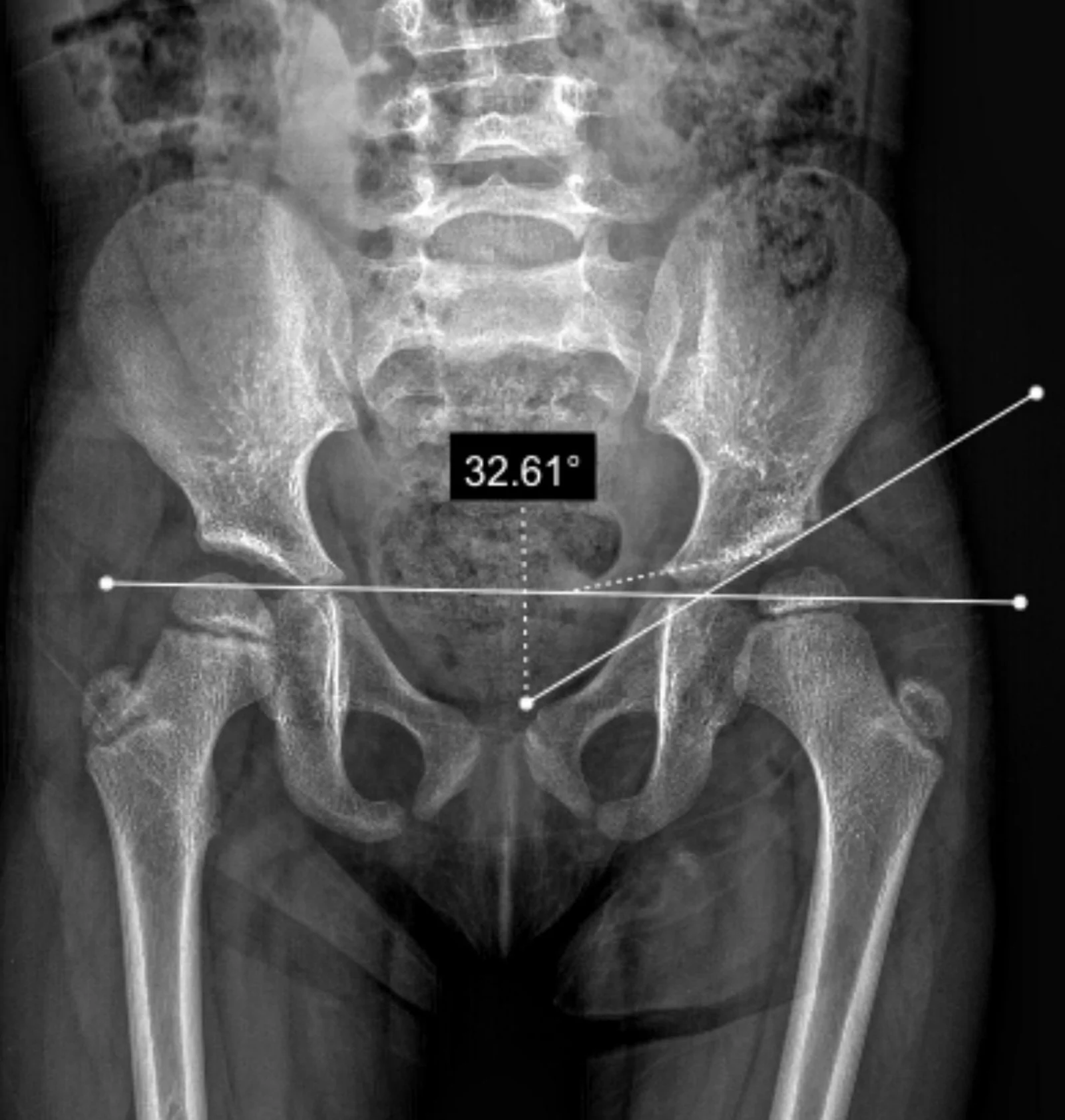
Radiographic measurement of the acetabular index at the 36-month follow-up of a patient with left developmental hip dislocation.

**Figure 7:**
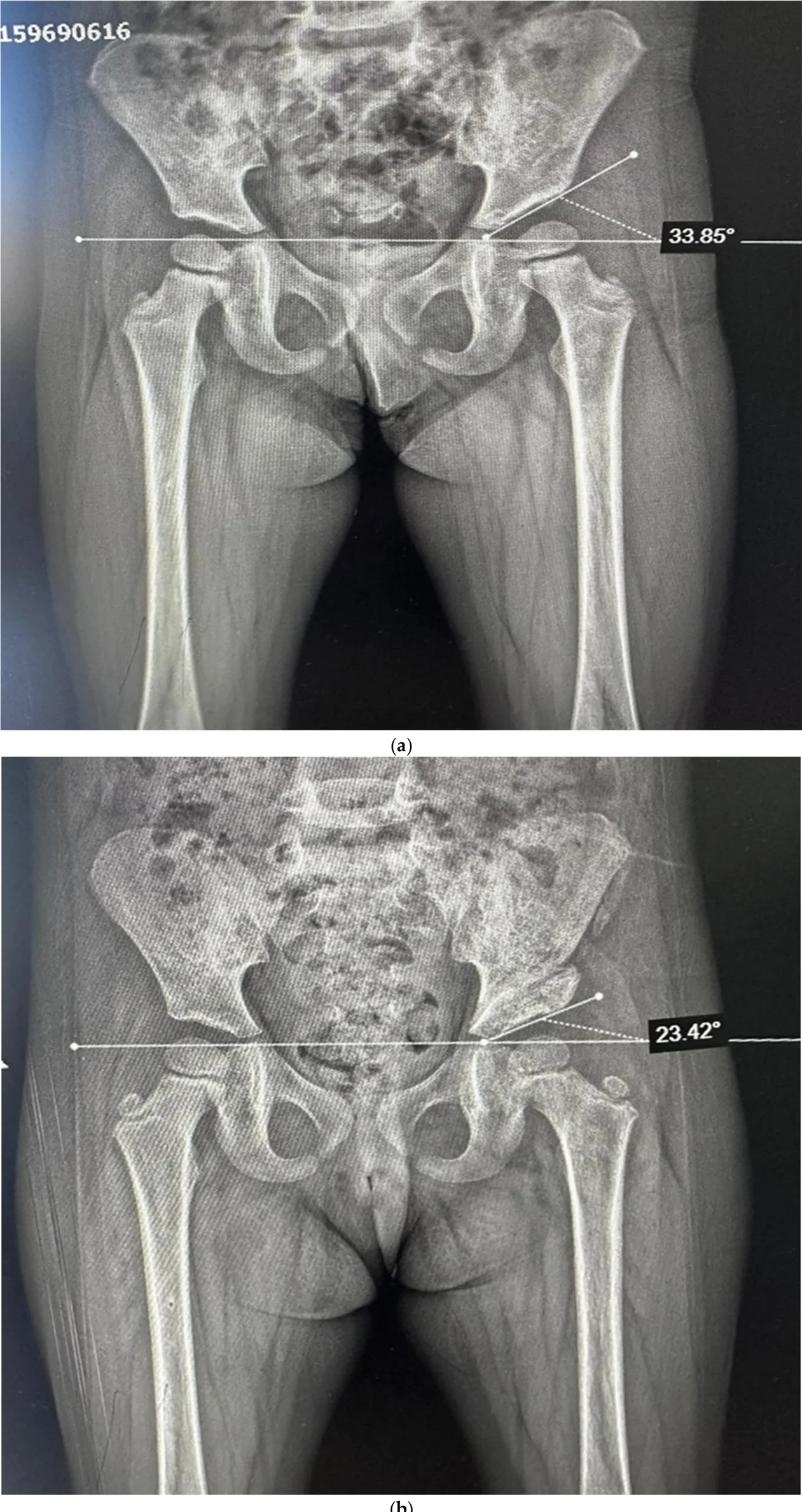
Residual dysplasia was observed during the 36-month acetabular index measurement of a child previously operated on for left developmental dysplasia of the hip (DDH). Surgical treatment (Pemberton osteotomy) was performed. (**a**): On the preoperative image, the acetabular index was 33.85°. (**b**): After surgery, the acetabular index was 23.42°.

**Table 1:** Descriptive Statistics of the Study Population.

Variable		n/Min	%/Max	Mean ± SD
AVN	Present	7	7.4%	
	Absent	88	92.6%	
Arthrography	Yes	50	52.6%	
	No	45	47.4%	
Osteotomy	Yes	15	15.8%	
	No	80	84.2%	
Tönnis Grade	Grade 1	0	—	
	Grade 2	7	7.4%	
	Grade 3	39	41.1%	
	Grade 4	49	51.6%	
Side	Right	48	50.5%	
	Left	47	49.5%	
Reduction Type	Open	63	66.3%	
	Closed	32	33.7%	
Age at Surgery (months)		6	24	12.83 ± 5.125
Follow-up (months)		25	60	37.78 ± 8.072
AI 6-month (°)		24	48	38.31 ± 4.470
AI 12-month (°)		20	45	31.59 ± 4.281
AI 36-month (°)		16	40	23.65 ± 6.026

AI = Acetabular Index; AVN = Avascular Necrosis; SD = Standard Deviation. For categorical variables, we report n and %; for continuous variables, we report Min, Max, Mean, and SD.

**Table 2:** Comparison of AVN and Osteotomy by Reduction Type.

Variable	Open n (%)	Closed n (%)	*p* (all)
AVN			0.091
Present	7 (11.1%)	0 (0.0%)	
Absent	56 (88.9%)	32 (100.0%)	
Osteotomy			0.081
Yes	13 (20.6%)	2 (6.3%)	
No	50 (79.4%)	30 (93.7%)	

Fisher’s exact test was used for all comparisons. Column percentages represent within-group rates. *p*-values indicate statistical significance (*p* < 0.05).

**Table 3:** Acetabular Index by Tönnis Grade (One-Way ANOVA).

AI Measure	Grade 2 (n = 7) $\bar{X}$ ± SD	Grade 3 (n = 39) $\bar{X}$ ± SD	Grade 4 (n = 49) $\bar{X}$ ± SD	Post Hoc (LSD)	*p*
6-month AI	34.00 ± 4.163	39.31 ± 4.578	38.12 ± 4.101	2 < 3 and 4	0.013
12-month AI	28.29 ± 3.498	31.41 ± 3.537	32.20 ± 4.839	—	0.071
36-month AI	19.57 ± 3.409	22.46 ± 5.389	25.18 ± 6.379	4 > 2 and 3	0.018

AI = Acetabular Index; $\bar{X}$ = Mean; SD = Standard Deviation.

**Table 4:** AI at 6 and 12 Months by Osteotomy Status.

AI Measure	Osteotomy (n = 15) $\bar{X}$ ± SD	No Osteotomy (n = 80) $\bar{X}$ ± SD	*p*
6-month AI	39.20 ± 4.554	38.14 ± 4.463	0.401
12-month AI	36.07 ± 4.234	30.75 ± 3.757	<0.001

AI = Acetabular Index; Independent-sample *t*-test. $\bar{X}$ = Mean; SD = Standard Deviation.

## Data Availability

The data supporting the findings of this study are available from the corresponding author upon reasonable request. The data are not publicly available due to patient privacy and institutional restrictions.
